# Supplementary material for: Mental health knowledge, stigma towards mental illness, and help-seeking among adolescents in secondary schools in Kampala, Uganda
Source: Glob Ment Health (Camb). 2026 Apr 6;13:e75. doi: 10.1017/gmh.2026.10194 (PMC13112280; doi:10.1017/gmh.2026.10194)
Supplement: Nantumbwe et al. supplementary material [file S2054425126101940sup001.docx]

**Appendix**

***Supplementary Tables***

**Supplementary Table S1:** Adolescent Mental Health Literacy Questionnaire (AMHLQ)

|  | Strongly agree | | Agree | | Neither agree nor Disagree | | Disagree | | Strongly disagree | |
| --- | --- | --- | --- | --- | --- | --- | --- | --- | --- | --- |
|  | n | % | n | % | n | % | n | % | n | % |
| 1. Talking over problems with someone close to me contributes to a good mental health. | 531 | 59.7% | 218 | 24.5% | 56 | 6.3% | 45 | 5.1% | 39 | 4.4% |
| 2. Doing something enjoyable contributes to a good mental health. | 619 | 69.6% | 186 | 20.9% | 42 | 4.7% | 28 | 3.1% | 14 | 1.6% |
| 3. A person with depression feels very miserable | 513 | 57.7% | 278 | 31.3% | 54 | 6.1% | 24 | 2.7% | 20 | 2.2% |
| 4. A person with schizophrenia may see and hear things that nobody else sees and hears | 261 | 29.4% | 181 | 20.4% | 374 | 42.1% | 17 | 1.9% | 56 | 6.3% |
| 5. Drug addiction may cause mental disorders | 616 | 69.4% | 191 | 21.5% | 53 | 6.0% | 9 | 1.0% | 19 | 2.1% |
| 6. Mental disorders affect people's thoughts | 630 | 70.9% | 198 | 22.3% | 39 | 4.4% | 13 | 1.5% | 9 | 1.0% |
| 7. Changes in brain function may lead to the onset of mental disorders. | 361 | 40.6% | 266 | 29.9% | 202 | 22.7% | 27 | 3.0% | 33 | 3.7% |
| 8. Highly stressful situations may cause mental disorders | 522 | 58.7% | 245 | 27.6% | 73 | 8.2% | 35 | 3.9% | 13 | 1.5% |
| 9. Sleeping well contributes to good mental health. | 664 | 74.7% | 153 | 17.2% | 29 | 3.3% | 29 | 3.3% | 14 | 1.6% |
| 10. Physical exercise contributes to good mental health. | 642 | 72.2% | 167 | 18.8% | 46 | 5.2% | 18 | 2.0% | 16 | 1.8% |
| 11. A balanced diet contributes to good mental health. | 578 | 65.0% | 185 | 20.8% | 74 | 8.3% | 37 | 4.2% | 15 | 1.7% |
| 12. If I had a mental disorder, I would seek my relatives' help. | 186 | 20.9% | 203 | 22.8% | 167 | 18.8% | 164 | 18.4% | 169 | 19.0% |
| 13. A person with anxiety disorder may panic in situations that she/he fears | 490 | 55.1% | 264 | 29.7% | 102 | 11.5% | 13 | 1.5% | 20 | 2.2% |
| 14. The symptom's length is one of the important criteria for the diagnosis of a mental disorder. | 159 | 17.9% | 174 | 19.6% | 440 | 49.5% | 28 | 3.1% | 88 | 9.9% |
| 15. One of the symptoms of depression is the loss of interest or pleasure in most things. | 490 | 55.1% | 227 | 25.5% | 102 | 11.5% | 28 | 3.1% | 42 | 4.7% |
| 16. Alcohol use may cause mental disorders | 520 | 58.5% | 223 | 25.1% | 96 | 10.8% | 31 | 3.5% | 19 | 2.1% |
| 17. A person with anxiety disorder avoids situations that may cause her/his distress | 351 | 39.5% | 250 | 28.1% | 184 | 20.7% | 58 | 6.5% | 46 | 5.2% |
| 18. People with schizophrenia usually have delusions (e.g., they may believe they are constantly being followed and observed) | 301 | 33.9% | 171 | 19.2% | 309 | 34.8% | 32 | 3.6% | 76 | 8.5% |
| 19. If someone close to me had a mental disorder, I would encourage her/him to go to a doctor | 491 | 55.2% | 223 | 25.1% | 86 | 9.7% | 46 | 5.2% | 43 | 4.8% |
| 20. If I had a mental disorder, I would seek professional help from a psychologist's AND/OR psychiatrist's help | 485 | 54.6% | 207 | 23.3% | 88 | 9.9% | 58 | 6.5% | 51 | 5.7% |
| 21. If someone close to me had a mental disorder, I would encourage her/him to look for a psychologist. | 521 | 58.6% | 230 | 25.9% | 84 | 9.4% | 28 | 3.1% | 26 | 2.9% |
| 22. If a friend close to me developed a mental disorder, I would talk to a form teacher or another teacher | 237 | 26.7% | 228 | 25.6% | 176 | 19.8% | 125 | 14.1% | 123 | 13.8% |
| 23. If I had a mental disorder, I would seek friends' help. | 197 | 22.2% | 201 | 22.6% | 178 | 20.0% | 149 | 16.8% | 164 | 18.4% |
| 24. If a friend close to me developed a mental disorder, I would talk to her/parents. | 342 | 38.5% | 260 | 29.2% | 129 | 14.5% | 80 | 9.0% | 78 | 8.8% |
| 25. People with mental disorders come from families with little money | 82 | 9.2% | 77 | 8.7% | 205 | 23.1% | 193 | 21.7% | 332 | 37.3% |
| 26. If someone close to me had a mental disorder, I would offer him/her support | 467 | 52.5% | 265 | 29.8% | 95 | 10.7% | 28 | 3.1% | 34 | 3.8% |
| 27. If someone close to me had a mental disorder, I could not be of any assistance. | 77 | 8.7% | 65 | 7.3% | 164 | 18.4% | 229 | 25.8% | 354 | 39.8% |
| 28. Depression is not a true mental disorder | 124 | 14.0% | 101 | 11.4% | 239 | 26.9% | 168 | 18.9% | 256 | 28.8% |
| 29. Mental disorders don't affect people's behaviours | 76 | 8.5% | 48 | 5.4% | 103 | 11.6% | 249 | 28.0% | 413 | 46.5% |
| 30. Only adults have mental disorders | 40 | 4.5% | 29 | 3.3% | 95 | 10.7% | 224 | 25.2% | 501 | 56.4% |
| 31. The sooner mental disorders are identified and treated, the better | 645 | 72.6% | 141 | 15.9% | 63 | 7.1% | 22 | 2.5% | 18 | 2.0% |
| 32. If someone close to me had a mental disorder, I would listen to her/him without judging or criticising. | 550 | 61.9% | 196 | 22.0% | 57 | 6.4% | 44 | 4.9% | 42 | 4.7% |
| 33. Mental disorders don't affect people's feelings | 91 | 10.2% | 51 | 5.7% | 140 | 15.7% | 224 | 25.2% | 383 | 43.1% |

**Supplementary Table S2:** Prejudice towards people with Mental Illness Turkish version (PPMI-TR)

|  | Strongly Agree | | Moderately Agree | | Agree | | Neither Agree nor Disagree | | Disagree | | Moderately Disagree | | Strongly Disagree | |
| --- | --- | --- | --- | --- | --- | --- | --- | --- | --- | --- | --- | --- | --- | --- |
|  | n | % | n | % | n | % | n | % | n | % | n | % | n | % |
| 1. I would find it hard to talk to someone who has a mental illness. | 210 | 23.6% | 238 | 26.8% | 97 | 10.9% | 83 | 9.3% | 111 | 12.5% | 44 | 4.9% | 106 | 11.9% |
| 2. I would be just as happy to invite a person with mental illness into my home as I would anyone else. | 199 | 22.4% | 179 | 20.1% | 107 | 12.0% | 147 | 16.5% | 116 | 13.0% | 39 | 4.4% | 102 | 11.5% |
| 3. I would feel relaxed if I had to talk to someone who was mentally ill. | 186 | 20.9% | 145 | 16.3% | 95 | 10.7% | 174 | 19.6% | 125 | 14.1% | 29 | 3.3% | 135 | 15.2% |
| 4. I am not scared of people with mental illness. | 265 | 29.8% | 133 | 15.0% | 113 | 12.7% | 123 | 13.9% | 92 | 10.4% | 27 | 3.0% | 135 | 15.2% |
| 5. In general, it is easy to interact with someone who has a mental illness. | 122 | 13.7% | 119 | 13.4% | 83 | 9.3% | 187 | 21.0% | 166 | 18.7% | 54 | 6.1% | 158 | 17.8% |
| 6. It is best to avoid people who have a mental illness. | 84 | 9.4% | 74 | 8.3% | 45 | 5.1% | 138 | 15.5% | 210 | 23.6% | 59 | 6.6% | 279 | 31.4% |
| 7. I would feel unsafe being around someone who is mentally ill. | 159 | 17.9% | 124 | 13.9% | 112 | 12.6% | 138 | 15.5% | 127 | 14.3% | 73 | 8.2% | 156 | 17.5% |
| 8. The behaviour of people with mental illness is unpredictable. | 448 | 50.4% | 86 | 9.7% | 150 | 16.9% | 101 | 11.4% | 33 | 3.7% | 19 | 2.1% | 52 | 5.8% |
| 9. The behaviour of people with mental illness is just as predictable as that of people who are mentally healthy. | 100 | 11.3% | 71 | 8.0% | 55 | 6.2% | 167 | 18.8% | 162 | 18.2% | 45 | 5.1% | 288 | 32.4% |
| 10. In general, you cannot predict how people with mental illness will behave. | 404 | 45.4% | 94 | 10.6% | 142 | 16.0% | 106 | 11.9% | 47 | 5.3% | 30 | 3.4% | 66 | 7.4% |
| 11. People with mental illness often do unexpected things. | 437 | 49.2% | 98 | 11.0% | 187 | 21.0% | 93 | 10.5% | 21 | 2.4% | 12 | 1.3% | 41 | 4.6% |
| 12. I usually find people with mental illness to be consistent in their behaviour. | 148 | 16.6% | 120 | 13.5% | 77 | 8.7% | 278 | 31.3% | 100 | 11.2% | 41 | 4.6% | 125 | 14.1% |
| 13. People with mental illness behave in ways that are foreseeable. | 172 | 19.3% | 84 | 9.4% | 80 | 9.0% | 260 | 29.2% | 100 | 11.2% | 41 | 4.6% | 152 | 17.1% |
| 14. People who are mentally ill should be free to make their own decisions. | 167 | 18.8% | 73 | 8.2% | 63 | 7.1% | 117 | 13.2% | 120 | 13.5% | 49 | 5.5% | 300 | 33.7% |
| 15. People who are mentally ill should be allowed to live their life any way they want. | 116 | 13.0% | 86 | 9.7% | 58 | 6.5% | 116 | 13.0% | 138 | 15.5% | 68 | 7.6% | 307 | 34.5% |
| 16. Society does not have a right to limit the freedom of people with mental illness. | 310 | 34.9% | 113 | 12.7% | 121 | 13.6% | 100 | 11.2% | 63 | 7.1% | 62 | 7.0% | 120 | 13.5% |
| 17. People who are mentally ill are avoiding the difficulties of everyday life. | 207 | 23.3% | 110 | 12.4% | 71 | 8.0% | 219 | 24.6% | 77 | 8.7% | 56 | 6.3% | 149 | 16.8% |
| 18. People who develop mental illness are genetically inferior to other people. | 137 | 15.4% | 94 | 10.6% | 60 | 6.7% | 246 | 27.7% | 94 | 10.6% | 66 | 7.4% | 192 | 21.6% |
| 19. People with mental illness do not deserve our sympathy. | 48 | 5.4% | 27 | 3.0% | 19 | 2.1% | 92 | 10.3% | 125 | 14.1% | 57 | 6.4% | 521 | 58.6% |

**Supplementary Table S3:** Self-identification as having a mental illness (SELF-I)

| Statement | Don’t Agree at All | Do Not Agree | Undecided | Agree | Agree Completely |
| --- | --- | --- | --- | --- | --- |
| 1. Current issues I am facing could be the first signs of a mental illness | 37.5% | 16.7% | 22.3% | 14.8% | 8.7% |
| 2. The thought of myself having a mental illness seems doubtful to me | 23.9% | 16.3% | 23.6% | 18.2% | 18.0% |
| 3. I could be the type of person that is likely to have a mental illness | 46.2% | 15.6% | 18.4% | 10.5% | 9.4% |
| 4. I see myself as a person that is mentally healthy and emotionally stable | 14.9% | 10.9% | 18.6% | 17.2% | 38.3% |
| 5. I am mentally stable; I do not have a mental health problem | 10.7% | 8.1% | 16.9% | 15.1% | 49.2% |

**Supplementary Table S4:** Correlations

Correlation between mental health knowledge, stigma, prejudice and self-identification

| **Variable** | **MAKS score** | **AMHLQ score** | **RIBS score** | **PPMI-TR score** |
| --- | --- | --- | --- | --- |
| **MAKS score** | 1.00 |  |  |  |
| Sig. (2-tailed) |  |  |  |  |
| **AMHLQ score** | .290** |  |  |  |
| Sig. (2-tailed) | .000 |  |  |  |
| **RIBS score** | .166** | .135** |  |  |
| Sig. (2-tailed) | .000 | .000 |  |  |
| **PPMI-TR score** | -.106** | -.071* | -.428** |  |
| Sig. (2-tailed) | .002 | .034 | .000 |  |
| **Self-I** | .030 | .071* | -.116** | .085* |
| Sig. (2-tailed) | .366 | .034 | .001 | .011 |

**. Correlation is significant at the 0.01 level (2-tailed).

*. Correlation is significant at the 0.05 level (2-tailed).
